# Investigating gene methylation signatures for fetal intolerance prediction

**DOI:** 10.1371/journal.pone.0250032

**Published:** 2021-04-22

**Authors:** Yu-Hang Zhang, Zhandong Li, Tao Zeng, Lei Chen, Hao Li, Margarita Gamarra, Romany F. Mansour, José Escorcia-Gutierrez, Tao Huang, Yu-Dong Cai

**Affiliations:** 1 School of Life Sciences, Shanghai University, Shanghai, China; 2 Channing Division of Network Medicine, Brigham and Women’s Hospital, Harvard Medical School, Boston, MA, United States of America; 3 College of Food Engineering, Jilin Engineering Normal University, Changchun, China; 4 Bio-Med Big Data Center, CAS Key Laboratory of Computational Biology, Shanghai Institute of Nutrition and Health, University of Chinese Academy of Sciences, Chinese Academy of Sciences, Shanghai, China; 5 College of Information Engineering, Shanghai Maritime University, Shanghai, China; 6 Department of Computational Science and Electronic, Universidad de la Costa, CUC, Barranquilla, Colombia; 7 Department of Mathematics, Faculty of Science, New Valley University, El-Kharga, Egypt; 8 Electronic and Telecommunicacions Program, Universidad Autónoma del Caribe, Barranquilla, Colombia; 9 CAS Key Laboratory of Tissue Microenvironment and Tumor, Shanghai Institute of Nutrition and Health, University of Chinese Academy of Sciences, Chinese Academy of Sciences, Shanghai, China; University of Mississippi Medical Center, UNITED STATES

## Abstract

Pregnancy is a complicated and long procedure during one or more offspring development inside a woman. A short period of oxygen shortage after birth is quite normal for most babies and does not threaten their health. However, if babies have to suffer from a long period of oxygen shortage, then this condition is an indication of pathological fetal intolerance, which probably causes their death. The identification of the pathological fetal intolerance from the physical oxygen shortage is one of the important clinical problems in obstetrics for a long time. The clinical syndromes typically manifest five symptoms that indicate that the baby may suffer from fetal intolerance. At present, liquid biopsy combined with high-throughput sequencing or mass spectrum techniques provides a quick approach to detect real-time alteration in the peripheral blood at multiple levels with the rapid development of molecule sequencing technologies. Gene methylation is functionally correlated with gene expression; thus, the combination of gene methylation and expression information would help in screening out the key regulators for the pathogenesis of fetal intolerance. We combined gene methylation and expression features together and screened out the optimal features, including gene expression or methylation signatures, for fetal intolerance prediction for the first time. In addition, we applied various computational methods to construct a comprehensive computational pipeline to identify the potential biomarkers for fetal intolerance dependent on the liquid biopsy samples. We set up qualitative and quantitative computational models for the prediction for fetal intolerance during pregnancy. Moreover, we provided a new prospective for the detailed pathological mechanism of fetal intolerance. This work can provide a solid foundation for further experimental research and contribute to the application of liquid biopsy in antenatal care.

## Introduction

Pregnancy is a complicated and long procedure during one or more offspring development inside a woman [1, 2]. Various pathological syndromes and severe situations may occur during pregnancy [3–5]. Fetal intolerance, which is also known as fetal distress, is one of the common but dangerous situations during birth processes [6]. It generally refers to babies suffering from oxygen shortage during the birth processes [6–8]. A short period of oxygen shortage after birth is quite normal for most babies and does not threaten their health [7]. However, if babies have to suffer from a long period of oxygen shortage, then this condition is an indication of pathological fetal intolerance, which probably causes their death.

The identification of the pathological fetal intolerance from the physical oxygen shortage is one of the important clinical problems in obstetrics for a long time. The following five symptoms according to the clinical syndromes indicate that the baby may suffer from fetal intolerance [9–11]:1) high heart rate or tachycardia; 2) low heart rate or bradycardia; 3) irregular heart rates or arrhythmia; 4) lack of movement in the womb; and 5) stool found in the amniotic fluid. For example, the alteration of the heart rate is quite normal for new born babies. However, the constant abnormal heart rate patterns and alterations strongly indicate pathological fetal intolerance [11]. Fetal intolerance is actually a quite severe disease and leads to the death of babies. The medical staff must save the baby after the manifestation of severe symptoms and try to find out an accurate and effective way to predict fetal intolerance (e.g., quick early diagnosis).

With the rapid development of molecule sequencing technologies, liquid biopsy [12–14] combined with high-throughput sequencing or mass spectrum techniques, provides a quick approach to detect real-time alteration in the peripheral blood at multiple levels (e.g., genomics [12], transcriptomics [13], and proteomics [14]). Various genetic variations, such as mutations in *IGF-II* and *H19*, have already been confirmed to participate in the pathogenesis of fetal intolerance [15]. The genomic methylation status has also been confirmed to be functionally correlated with fetal intolerance. In 2018, an independent study on the methylation status of *SLC9B1* has confirmed that such methylation pattern can actually predict the clinical outcome of potential pregnancy related to fetal intolerance [16]. Gene methylation is functionally correlated with gene expression. Thus, the combination of gene methylation and expression information would help in screening out the key regulators for the pathogenesis of fetal intolerance.

We combined gene methylation and expression features together and screened out the optimal features, including gene expression or methylation signatures for fetal intolerance prediction, for the first time. Moreover, we have applied various computational methods to construct a comprehensive computational pipeline to identify the potential biomarkers for fetal intolerance dependent on the liquid biopsy samples. We set up qualitative and quantitative computational models for the prediction for fetal intolerance during pregnancy. Furthermore, we provided a new prospective for the detailed pathological mechanism of fetal intolerance. This work can provide a solid foundation for further experimental research and contribute to the application of liquid biopsy in the antenatal care.

## Method

### Data

We downloaded the gene expression and methylation profiles of fetal intolerance from Gene Expression Omnibus (GEO, https://www.ncbi.nlm.nih.gov/geo/query/acc.cgi?acc=GSE107460) [16]. We extracted 22 fetal intolerance and 96 control samples with gene expression and methylation profiles from the original dataset. The expression levels of 15,505 genes were measured with Illumina HumanHT-12 V4.0 expression beadchip. The methylation data were measured with Illumina HumanMethylation450 BeadChip. The probes with missing values in more than 20% of the samples were removed. Thereafter, the remaining missing values were imputed with function impute.knn (K = 10) by using R package impute (https://bioconductor.org/packages/impute/). Lastly, 449,094 methylation probes were found. We would like to investigate the gene expression and methylation difference between fetal intolerance and control samples.

### SMOTE

The dataset we analyzed here has unbalanced numbers of positive and negative samples (i.e., 22 vs. 96). We first applied the synthetic minority oversampling technique (SMOTE) [17] to obtain a balanced data benefitting the classification model construction. SMOTE aims to iteratively produce new samples for the minor sample class (i.e., fetal intolerance samples) to ensure that the sample numbers of this minor sample class will be equivalent to that of the major one (i.e., control samples) when SMOTE is finished. In this study, the tool “SMOTE” in Weka is used to produce equivalent numbers of samples.

### Boruta feature filtering

Boruta feature filtering [18] can filter all features relevant to the target outputs on the basis of random forest (RF) in a wrapper manner. This algorithm recognizes important features by comparing the importance scores corresponding to the real and shuffled features. The following are the three main calculation steps for the Boruta approach: i) production of a new shuffled dataset by copying the training dataset and shuffling the feature values; ii) calculation of the importance score of each feature by training a RF classifier on the shuffled dataset; and iii) evaluation of the importance score of each feature in the original training dataset to retain the real features with remarkably higher importance scores than the shuffled features.

### Feature ranking and selection

#### Minimum redundancy maximum relevance

Minimum redundancy maximum relevance (mRMR) [19–22] holds two key assumptions: one is to select features with minimum redundancy among themselves; and the other one is to select features with maximum relevance with class labels. The mRMR filters informative features by selecting the features that simultaneously satisfy the minimum redundancy and maximum relevance measured by mutual information. These factors are important or informative to ensure that the following classification model can distinguish class labels (e.g., fetal intolerance or not in this work).

#### Incremental feature selection

Incremental feature selection (IFS) [23] can iteratively determine the optimal number of selected features with feature order. First, IFS selects a series of feature subsets from the mRMR ranked features. For example, the first selected feature subset consists of the top-ranked one feature, and the second one is composed of the top-ranked two features. In each training data consisting of features from each feature subset, IFS trains one classification model. The performance is evaluated in the 10-fold cross-validation [24]. Finally, IFS selects the feature subset with optimal performance as the optimum feature subset.

#### Classification algorithm

**RF.** RF [25–27] creates an assemble classification model consisting of several tree classifiers. The RF determines the predicted sample class/category by an aggregating vote from multiple tree classifiers (i.e., decision trees). The RF produces the final consensus results by averaging all decision trees’ predictions because a subtle difference exists between each decision tree. Accordingly, overfitting is avoided, and the model performance robustness is improved.

#### Support vector machine

The support vector machine (SVM) [28–31] is a classification model based on statistical learning theory. This model can map data samples to a given data class/category. SVM aims to transform the original data from a low-dimensional data space to a high-dimensional one by using a given kernel function (e.g., Gaussian kernel). Thereafter, the model can divide the data samples of each class/category by maximizing the data interval in the high-dimensional data space during training. Subsequently, the model further predicts/tests a new sample’ category depending on the interval where this new sample belongs. In this study, we use the sequence minimization optimization algorithm implemented in Weka software [32, 33] to create an SVM for a two-class classification model.

#### Rule learning classifier RIPPER

We used RIPPER [34] generating classification rules to classify the samples from different classes/categories. RIPPER can predict new data by learning the interpretable classification model in accordance with the IF–ELSE rules. Moreover, RIPPER can learn all rules for each sample class; it learns the rules for one class and then moves to learn the rules for the next class. Learning starts from the minority sample class and then to the second minority sample class until the dominant class. In this study, the “JRip” algorithm implemented in Weka software was used.

### Performance evaluation

In this study, a commonly used evaluation method, namely, the Matthew correlation coefficient [35–37] (MCC), is used to evaluate the prediction performance of each classification model within a 10-fold cross-validation. MCC has a value ranging between −1 and +1 and achieves +1 when the classification model has a good performance. In this study, we evaluate the two-class classification models. Thus, the MCC for binary problem is adopted as follows:

$$MCC=\frac{TP\times TN-FP\times FN}{\sqrt{\left(TP+FP)(TP+FN)(TN+FP)(TN+FN\right)}},$$ (1)

where *TP*, *TN*, *FP*, and *FN* are the number of true-positive, true-negative, false-positive, and false-negative samples, respectively. Furthermore, we also counted sensitivity (SN), specificity (SP) and accuracy (ACC) for each model to give a full evaluation.

## Results and discussion

**Fig 1:**
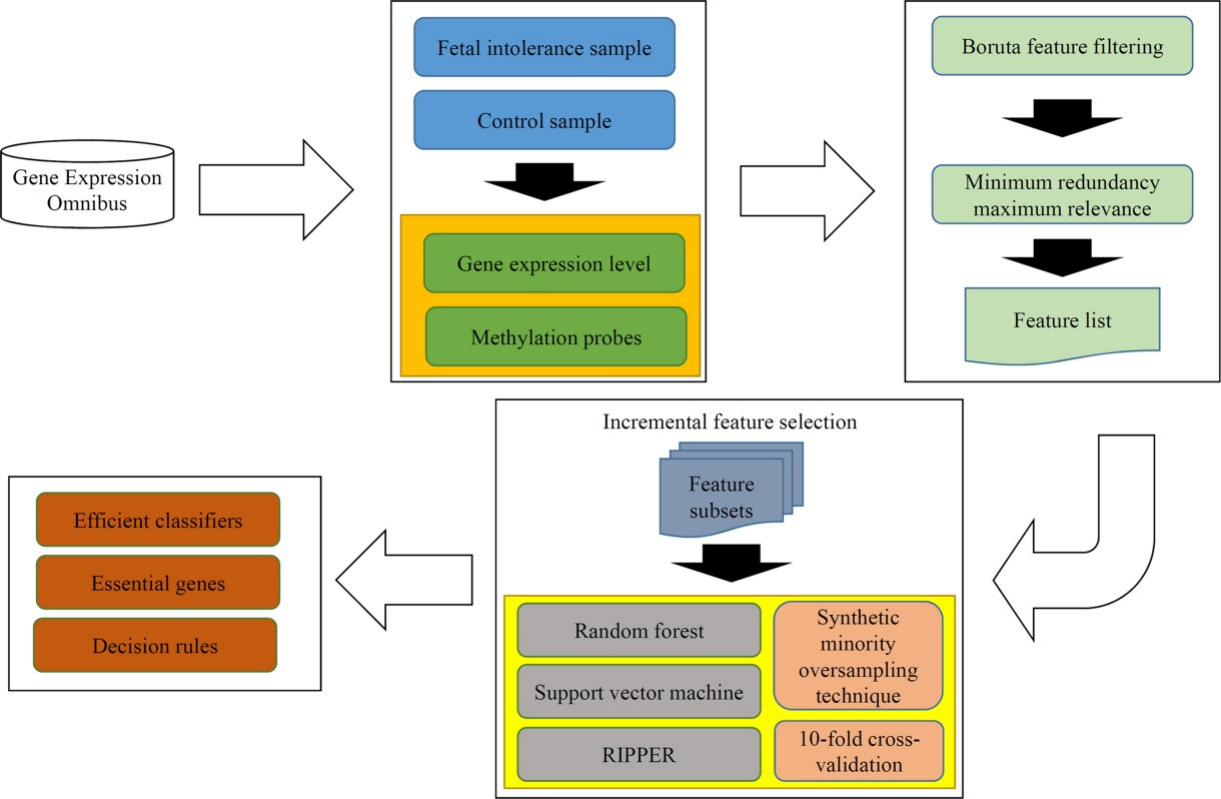
Whole procedures for analyzing the gene expression and methylation profiles of fetal intolerance. The original dataset is retrieved from Gene Expression Omnibus. Some feature selection methods (Boruta feature filtering and minimum redundancy maximum relevance) follow to analyze the dataset, resulting in a feature list. Incremental feature selection generates several feature subsets from the feature list, on each of which a model is built using one of three classification algorithms. Models are tested by 10-fold cross-validation using synthetic minority oversampling technique to tackle imbalanced problem. Finally, some efficient classifiers, essential genes and decision rules are obtained.

In this study, we adopted several advanced computational methods to analyze the gene expression and methylation profiles of fetal intolerance. The whole procedures are illustrated in **Fig 1**. This section gave the detailed results and performed the discussion on the results.

### Results of Boruta and mRMR

The dataset was first analyzed by Boruta to select key features. 15 relevant features were kept, which are provided in **S1 Table**. These features were further evaluated by mRMR method, a feature list was generated, which are also available in **S1 Table**.

### Selected features and classification models for distinguishing pregnant patients with or without fetal intolerance

**Fig 2:**
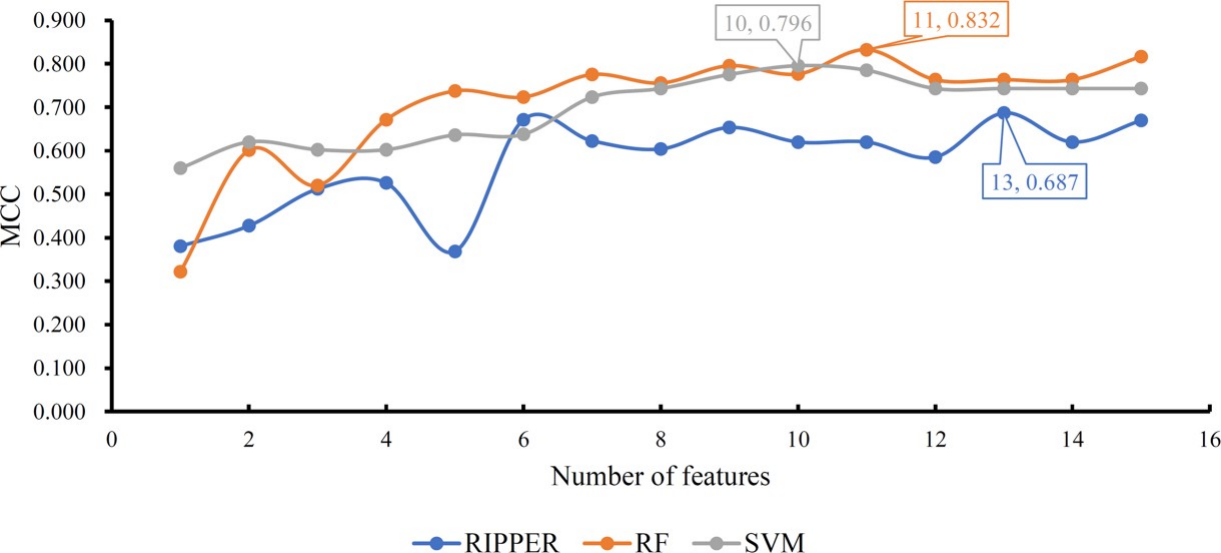
IFS curves based on the ordered features from RF, SVM, and RIPPER. The model with RF and top 11 features is the best, which produces the MCC of 0.832.

Of the obtained feature list, IFS method generated several feature subsets in a way that the top feature comprised the first feature subset, the top two features constituted the second feature subset, and so forth. Fifteen feature subsets were accessed. For each feature subset, a classification model was built using one of the three classification algorithms (RF, SVM and RIPPER). Each model was evaluated by 10-fold cross-validation. This procedure employed SMOTE to tackle the problem that control samples were much more than fetal intolerance sample. Obtained SNs, SP, ACCs and MCCs are listed in **Tables 1–3**. For an easy observation, we plotted a curve for the IFS results with each classification algorithm, as shown in **Fig 2**, in which MCC was set as Y-axis and the number of features was set as X-axis. For SVM, the highest MCC was 0.796, which was obtained by the top 10 features. Thus, we can build an optimum SVM model with these top 10 features. The other three measurements (SN, SP and ACC) of such model are listed in **Table 2**. The highest MCC was 0.832 for RF when top 11 features were adopted. Accordingly, an optimum RF model was built with these top 11 features. The SN, SP and ACC of this model are provided in **Table 1**. Evidently, the optimum RF model was superior to the optimum SVM model.

**Table 1:** IFS performance with RF and different top features.

Number of features	Sensitivity	Specificity	Accuracy	Matthew correlation coefficient
1	0.677	0.727	0.686	0.322
2	0.813	0.909	0.831	0.601
3	0.854	0.727	0.831	0.520
4	0.906	0.818	0.890	0.672
5	0.906	0.909	0.907	0.738
6	0.917	0.864	0.907	0.723
7	0.927	0.909	0.924	0.775
8	0.917	0.909	0.915	0.756
9	0.938	0.909	0.932	0.796
10	0.958	0.818	0.932	0.777
11	0.969	0.864	0.949	0.832
12	0.938	0.864	0.924	0.764
13	0.938	0.864	0.924	0.764
14	0.938	0.864	0.924	0.764
15	0.948	0.909	0.941	0.817

**Table 2:** IFS performance with SVM and different top features.

Number of features	Sensitivity	Specificity	Accuracy	Matthew correlation coefficient
1	0.938	0.591	0.873	0.560
2	0.875	0.818	0.864	0.620
3	0.885	0.773	0.864	0.603
4	0.885	0.773	0.864	0.603
5	0.885	0.818	0.873	0.636
6	0.865	0.864	0.864	0.638
7	0.917	0.864	0.907	0.723
8	0.927	0.864	0.915	0.743
9	0.927	0.909	0.924	0.775
10	0.938	0.909	0.932	0.796
11	0.948	0.864	0.932	0.785
12	0.927	0.864	0.915	0.743
13	0.927	0.864	0.915	0.743
14	0.927	0.864	0.915	0.743
15	0.927	0.864	0.915	0.743

**Table 3:** IFS performance with RIPPER and different top features.

Number of features	Sensitivity	Specificity	Accuracy	Matthew correlation coefficient
1	0.802	0.636	0.771	0.380
2	0.750	0.773	0.754	0.428
3	0.823	0.773	0.814	0.512
4	0.833	0.773	0.822	0.526
5	0.760	0.682	0.746	0.369
6	0.865	0.909	0.873	0.671
7	0.854	0.864	0.856	0.623
8	0.865	0.818	0.856	0.604
9	0.875	0.864	0.873	0.654
10	0.875	0.818	0.864	0.620
11	0.875	0.818	0.864	0.620
12	0.896	0.727	0.864	0.586
13	0.896	0.864	0.890	0.687
14	0.875	0.818	0.864	0.620
15	0.885	0.864	0.881	0.670

As RF and SVM are black-box algorithms, their classification principle is hard to understand. Thus, few insights can be obtained. In view of this, we further applied RIPPER in a similar way. The IFS performance is shown in **Table 3**, from which a curve was plotted, as illustrated in **Fig 2**. The best MCC was 0.687 when top 13 features were used. Thus, an optimum RIPPER model was set up with these features. Other three measurements of this model are listed in **Table 3**. Clearly, such model was inferior to the optimum RF and SVM models. However, some rules can be extracted from this model, which clearly displayed the classification procedures. Based on top 13 features, we obtained five rules via RIPPER, which are listed in **Table 4**.

**Table 4:** Decision rules generated by RIPPER on selected features.

Index	Condition	Result
Rule1	(cg23159165< = 0.4524) and (cg26222765< = 0.8847)	Fetal intolerance sample
Rule2	(cg04944931 < = 0.8437) and (cg00510160 < = 0.7120)	Fetal intolerance sample
Rule3	(cg19672271 > = 0.05775) and (cg00510160 < = 0.7221)	Fetal intolerance sample
Rule4	(cg04944931 < = 0.6588)	Fetal intolerance sample
Rule5	Others	Control sample

As previously mentioned, we presented various qualitative and quantitative novel computational approaches to distinguish pregnant patients with fetal intolerance from healthy pregnant women dependent on their personal blood gene expression and methylation profiles. We not only identified a group of effective genes with a specific gene expression or methylation pattern that contributes to the diagnosis of fetal intolerance but also attempted to set up a set of quantitative rules for accurate and interpretable prediction on the basis of our methods. All the predicted gene expression and methylation patterns and their quantitative rules have been confirmed by recent publications. The detailed analysis and discussion on the top-ranked genes and rules can be seen below.

### Optimal genes for fetal intolerance diagnosis and monitoring

Our newly presented computational methods identified fifteen methylation sites that are correlated with fetal intolerance and involved in five genes: *NHEDC1*, *COMTD1*, *DLGAP2*, *HEG1*, and *KIAA1875*.

The first gene (*NHEDC1*) with five methylation sites, also known as *SLC9B1*, has been widely reported to participate in the intracellular pH regulation in germ cells [38]. Such gene has been reported to have quite various biological effects with different methylation statuses [39, 40]. The abnormal methylation of such gene has been confirmed to participate in cell differentiation [39]. Such gene has already been reported as a typical biomarker for the clinical prediction of fetal intolerance [16], thereby validating the efficacy and accuracy of our prediction.

The second gene is *COMTD1*, which encodes an effective methyltransferase with O-methyltransferase activity [41–43]. No direct evidence confirmed that *COMTD1* can independently predict fetal intolerance; however, *COMTD1* in a mother’s blood is correlated with several congenital disorders, such as psychotic diseases and autism [44]. Given that congenital disorders are among the major inducements for fetal intolerance [45, 46], biomarkers are correlated with such gene to monitor this condition. Furthermore, COMTD1 has been confirmed to be detectable in the blood on the methylation level [43]. This finding confirms the potentials of such gene as an effective biomarker for fetal intolerance prediction and monitoring.

*DLGAP2* is the third gene encoding a specific membrane associated protein and has been widely reported to participate in the molecular organization of synapses and neuronal cell signaling [47, 48]. In 2019, an independent study confirmed that the methylation status of such gene in the blood can monitor the blood sugar level of mothers and maternal insulin sensitivity during pregnancy [49, 50]. Considering that the blood sugar level of mothers is also pathologically correlated with fetal intolerance [51, 52], such gene can be regarded as a potential biomarker during fetal intolerance monitoring and diagnosis.

*HEG1* is a quite effective regulator for the heart and vessels during the early developmental stage [53]. In 2019, a report confirmed that the abnormal methylation regulation on such gene may induce trophoblast invasion at the maternal–fetal interface, thereby inducing a high level of mothers’ psychological distress [54] and the abnormal development of fetal hearts [54, 55], even though not validated in human beings. Considering that fetal heart development has also been predicted to be correlated with fetal intolerance [56–58], *HEG1* can be regarded as an effective biomarker for fetal intolerance prediction. Gene *KIAA1875* known as *WDR97* has been reported as a blood biomarker with moderate functional annotations [59]. This gene has also been confirmed to be detectable at the epigenomic level in the blood [60]. Thus, *KIAA1875* may act as a quality control biomarker to measure the reliability of the samples, although no direct reports at present has confirmed its specific role in fetal intolerance prediction.

### Optimal rules for fetal intolerance diagnosis and monitoring

We set up a group of quantitative rules for diagnosing the fetal intolerance in clinical application in addition to above qualitative analysis. The four rules are used to evaluate the risk of pregnant mothers suffering from fetal intolerance. Five methylation sites with specific methylation tendency (hypermethylation or hypomethylation) contribute to the prediction of these rules. Among these methylation sites, two genes are annotated: *COMTD1* and *NHEDC1*. These genes have already been confirmed to be functionally correlated with fetal intolerance in the above analysis.

The hypermethylation of *NHEDC1* and the hypomethylation of *COMTD1* contribute to the identification of patients with fetal intolerance, thereby revealing the specific methylation tendency (hypermethylation or hypomethylation) from the rules. Recent studies have shown that the methylation of *NHEDC1* can indicate the onset of fetal intolerance [16], thereby supporting this prediction. *COMTD1* is functionally correlated with fetal intolerance, and its hypomethylation may cause abnormal congenital disorders [41], thereby inducing pathological fetal intolerance. Therefore, these quantitative rules contribute to the accurate prediction of fetal intolerance using blood samples.

## Conclusion

In summary, the optimal genes and rules we identified in this study have all been supported by recent publications. The efficacy and accuracy of our prediction have also been validated. The blood gene methylation profiling of certain effective biomarkers may be accurate and effective enough for the clinical monitoring of fetal intolerance during pregnancy by using our newly presented computational approaches. Therefore, this work may not only reveal several potential pathological factors for fetal intolerance but also set up a set of potential diagnostic standards (biomarkers and rules) for the clinical monitoring and diagnosis of fetal intolerance.

## Supporting information

S1 Table List of ranked features on the basis of mRMR. (DOCX)
