# Supplementary material for: Investigating gene methylation signatures for fetal intolerance prediction
Source: PLoS One. 2021 Apr 22;16(4):e0250032. doi: 10.1371/journal.pone.0250032 (PMC8062050; doi:10.1371/journal.pone.0250032)
Supplement: S1 Table — (DOCX) [file pone.0250032.s001.docx]

**S1 Table.** List of ranked features on the basis of mRMR

| **Rank** | **Feature** |
| --- | --- |
| 1 | cg05605371 |
| 2 | cg04944931 |
| 3 | cg19571721 |
| 4 | cg16901123 |
| 5 | cg06999381 |
| 6 | cg26222765 |
| 7 | cg23206461 |
| 8 | cg21197425 |
| 9 | cg23159165 |
| 10 | cg10364301 |
| 11 | cg00510160 |
| 12 | cg03440673 |
| 13 | cg19672271 |
| 14 | cg13067553 |
| 15 | cg14631276 |
